# Proposed U.S. regulation of gene-edited food animals is not fit for purpose

**DOI:** 10.1038/s41538-019-0035-y

**Published:** 2019-03-20

**Authors:** Alison L. Van Eenennaam, Kevin D. Wells, James D. Murray

**Affiliations:** 1 Department of Animal Science, University of California, Davis, CA USA; 2 Division of Animal Sciences, University of Missouri, Columbia, MO USA; 3 Department of Population Health and Reproduction, School of Veterinary Medicine, University of California, Davis, CA USA

**Keywords:** Metabolic engineering, Agriculture

## Abstract

Dietary DNA is generally regarded as safe to consume, and is a routine ingredient of food obtained from any living organism. Millions of naturally-occurring DNA variations are observed when comparing the genomic sequence of any two healthy individuals of a given species. Breeders routinely select desired traits resulting from this DNA variation to develop new cultivars and varieties of food plants and animals. Regulatory agencies do not evaluate these new varieties prior to commercial release. Gene editing tools now allow plant and animal breeders to precisely introduce useful genetic variation into agricultural breeding programs. The U.S. Department of Agriculture (USDA) announced that it has no plans to place additional regulations on gene-edited plants that could otherwise have been developed through traditional breeding prior to commercialization. However, the U.S. Food and Drug Administration (FDA) has proposed mandatory premarket new animal drug regulatory evaluation for all food animals whose genomes have been intentionally altered using modern molecular technologies including gene editing technologies. This runs counter to U.S. biotechnology policy that regulatory oversight should be triggered by unreasonable risk, and not by the fact that an organism has been modified by a particular process or technique. Breeder intention is not associated with product risk. Harmonizing the regulations associated with gene editing in food species is imperative to allow both plant and animal breeders access to gene editing tools to introduce useful sustainability traits like disease resistance, climate adaptability, and food quality attributes into U.S. agricultural breeding programs.

The reason some cattle grow horns whereas others do not (Fig. 1), and a Granny Smith looks different from a Red Delicious apple is due to selection by breeders on naturally-occurring variations in genomic DNA sequences. Technically, these variations are known as alleles and result from changes, or variations, in the DNA sequence caused by mutations. There are literally millions of naturally-occurring DNA variations between any two healthy individuals of a given species. These variations are the reason genetic tests like “23andMe™” can identify family members and lineages; we share more unique alleles, or mutations, with our close relatives than we do with unrelated individuals.

**Fig. 1:**
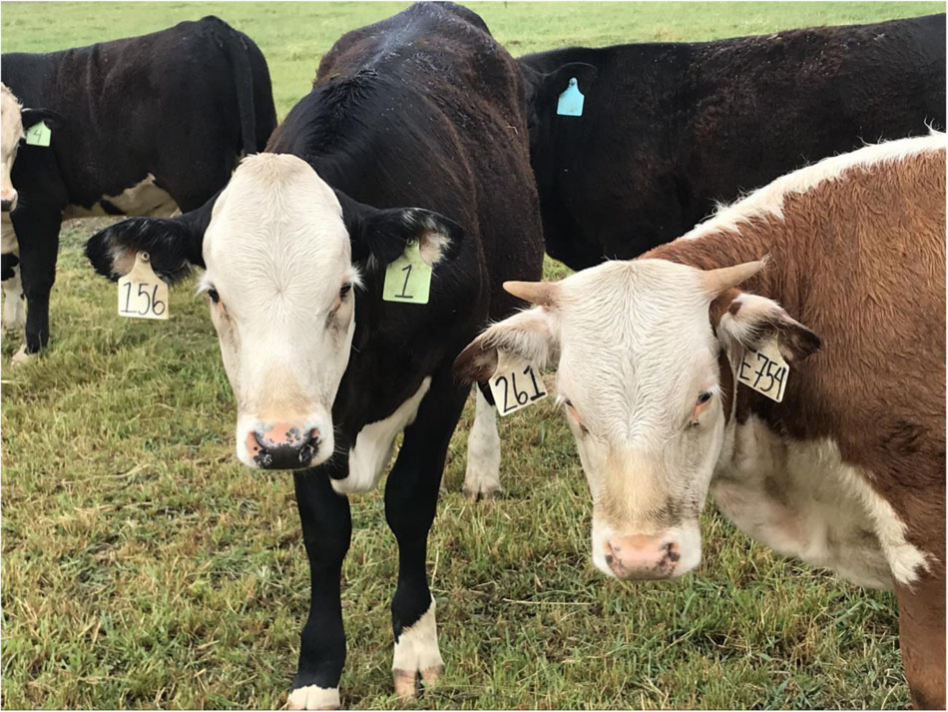
Hornless offspring of a gene edited bull alongside a horned control cow. The reason cattle differ in their coat color, or whether or not they grow horns, is due to naturally occurring DNA sequence variations in their genome. These DNA sequence variations can also be introduced using gene editing as occurred with this black cow (#1) who inherited a gene-edited *POLLED* DNA sequence variant (P_C_ allele) from her father, and as a result she is genetically hornless. Photo by Alison Van Eenennaam, University of California, Davis

To put this in perspective, one study of whole genome sequence data from 2703 individual cattle in the 1000 Bull Genomes Project revealed more than 86.5 million differences (variants) between different breeds of cattle. These variants included 2.5 million insertions and deletions of one, or more, base pairs of DNA, and 84 million single nucleotide variants, where one of the four nucleotides making up DNA (A, C, G, T) had been changed to a different one.^1^ A small fraction of these mutations have been selected by breeders owing to their beneficial effects on characteristics of agronomic importance. None of these naturally-occurring variants are known to produce ill effects on the consumers of milk or beef products. In fact, every meal we have ever consumed is genetically distinct from every other meal in terms of genomic DNA sequences. Genetic variation per se does not pose a unique hazard as it relates to food safety. All non-processed foods harbor DNA as a natural component and that DNA is different in every individual of every food species (both plants and animals).

Variations in the DNA between individuals result in differences in appearance, known as phenotypes. The observable characteristics of each selection candidate (individual that may be selected for breeding), resulting from the interaction of its genotype with the environment, are recorded during routine phenotypic evaluations. So-called “off-types” that deviate from the desired characteristics are identified and not used for breeding purposes. Breeders select only the most viable, productive, and healthy individuals to be parents of the next generation. In the words of one animal geneticist,^2^ “For millennia, animal breeders have performed what amounts to a mega-scale, phenotype-driven mutagenesis screen.”

Although plants and animals produced from conventional breeding methods are routinely evaluated for changes in productivity, reproductive efficiency, reactions to disease, and quality characteristics, they are not routinely evaluated for unintended effects at the molecular level.^3^ Regulatory agencies do not evaluate new conventionally-bred varieties for health and environmental safety prior to commercial release. Selection for more productive and resilient plant and animal varieties has been an incredibly important component of improving yield while resulting in a decreased environmental footprint per unit of food production. Since 1960, global livestock productivity has increased 20–30%,^4^ due in large part to genetic improvements resulting from selection.^5,6^

Traditional plant breeding programs have used mutagenic chemicals like ethyl methane sulfonate or fast neutron irradiation to induce mutations, or changes, in DNA sequences at random loci throughout the genome in an attempt to generate novel trait variation since the 1930s.^7^ This increases the genetic variation that is available to breeding programs.^8^ The widespread use of mutation techniques in plant breeding programs throughout the world has generated thousands of novel crop varieties in hundreds of crop species. There are over 3200 mutant varieties from 214 different plant species officially released in more than 70 countries as referenced in the Mutant Varieties Database (https://mvd.iaea.org/). There are no unique regulatory guidelines or tracking required for these varieties, and there do not appear to be any documented examples in which mutant varieties were removed from the market due to unintended or unexpected adverse incidents.^3^

Most crops naturally produce allergens, toxins, or other anti-nutritional substances. In their 2004 report, the National Research Council noted some rare safety issues that have been associated with conventional plant breeding,^3^ such as allergens in Kiwi fruit,^9^ or high levels of solanine in potatoes.^10,11^ Figure 2 represents the magnitude of biological variation that exists between different individuals, and sources of technical variation that can occur due to differences in sequencing platform, errors, and bioinformatics based on literature estimates.^1,7,8,12,13^

**Fig. 2:**
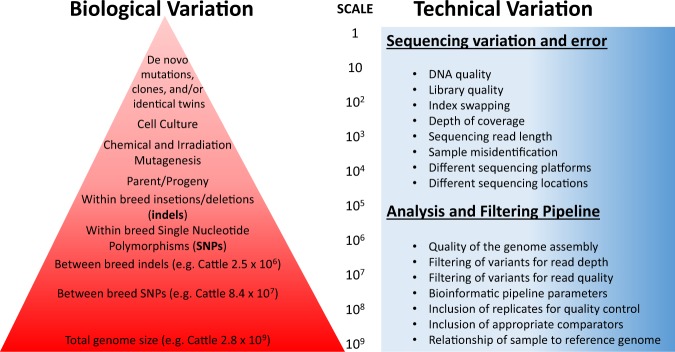
Visual representation of the magnitude of biological variation that exists between different individuals, and sources of technical variation that can occur due to differences in sequencing platform, errors, and bioinformatics. Due to the fact that plant breeders often use self-fertilization to develop cultivars prior to release, plants tend to have much less variation between individuals of the same cultivar than is seen between two out-crossing cattle of the same breed. The quantification and detection of sequence variation is exquisitely sensitive to differences in the sequencing and bioinformatics pipeline

Gene editing is a technique that can be used to introduce useful genetic variations into breeding programs. It involves the use of enzymes that cut DNA at a specific sequence (site-specific nucleases e.g., CRISPR-Cas9), thereby introducing a break into the DNA at a targeted location. Depending upon how that break is naturally mended by the DNA repair mechanisms in the cell, genetic variations can be introduced that range from nucleotide deletions, or insertions, to substitutions of one nucleotide for another. Gene editing opens up new opportunities to introduce targeted genetic variations and develop breeds and lines where undesirable traits are precisely knocked out, or eliminated, and desirable traits are precisely knocked in to genomes.^14^ The phenotype that results from such alterations will depend upon which gene was targeted. For example, plant breeders have already used these tools to improve sustainability traits including disease resistance, drought and salt tolerance, and product quality.^15^

In March 2018, the USDA announced that it had no plans to evaluate gene-edited plants for health and environmental safety prior to commercial release if they could otherwise have been developed through traditional breeding, so long as the crop is not a plant pest or developed using plant pests. Under this ruling, genetic deletions, single base-pair substitutions and the insertion (introgression) of nucleotide sequences from related plants that could potentially have come about through crossbreeding, are all outside the scope of USDA regulation.^16^

In early 2017, the United States Food and Drug Administration (FDA) released its updated draft “Guidance for Industry #187” entitled, “Regulation of Intentionally Altered Genomic DNA in Animals”.^17^ This guidance proposes to regulate all food animals whose genomes have been intentionally altered using modern molecular technologies including gene editing technologies as veterinary drugs. This is the approach that the FDA has used to regulate genetically engineered (GE), also known as bioengineered, animals since 2009. Regrettably, this approach has not enabled the use of any technology that utilizes recombinant DNA (rDNA) in food animal breeding programs. As of March 2019, no food from an animal that has an intentional genotype produced from any technology that utilized rDNA has ever reached the U.S. market.

To understand how this situation arose, some background is required. The first paper documenting the production of transgenic GE food animals was published in 1985.^18^ In 1986, the White House, Office of Science and Technology Policy (OSTP) published the Coordinated Framework for Regulation of Biotechnology.^19^ This document states, “This framework has sought to distinguish between those organisms that require a certain level of federal review and those that do not. This follows a traditional approach to regulation. Within agriculture, for example, introductions of new plants, animals and microorganisms have long occurred routinely with only some of those that are not native or are pathogenic requiring regulatory approval.” The document goes on to clarify that regulatory review should be confined to organisms deliberately formed to contain an intergeneric combination of genetic material from sources in different genera, subsequently known as “transgenic” organisms.

In the 2009 FDA Guidance for Industry #187 entitled, “Regulation of Genetically Engineered Animals Containing Heritable rDNA Constructs”,^20^ the FDA announced its intent to regulate all GE animals modified by rDNA techniques, including the entire lineage of animals that contain the modification, under the new animal drug provisions of the Federal Food, Drug, and Cosmetic Act (FD&C Act). In that act, a new animal drug is defined as “an article (other than food) intended to affect the structure or any function of the body of … animals.” The FDA clarified that they considered the rDNA construct in a GE animal to be the drug, not the GE animal itself. And although the FDA’s regulatory evaluation is based on attributes of the product (the GE animal), the method used to produce the genetic change, that is rDNA versus other breeding methods, is the trigger for regulatory oversight. In other words, the FDA triggers regulatory oversight based on the process designed to produce the GE animal, not on the basis of the specific characteristics of the animal or its food products (milk, meat or eggs).

This was already a departure from the approach that was outlined by OSTP in the 1992 policy announcement.^21^ There it is stated that, “Exercise of oversight in the scope of discretion afforded by statute should be based on the risk posed by the introduction and should not turn on the fact that an organism has been modified by a particular process or technique”. Additionally, it was clarified that “(O)versight will be exercised only where the risk posed by the introduction is unreasonable, that is, when the value of the reduction in risk obtained by additional oversight is greater than the cost thereby imposed.”

The prohibitive cost, and open-ended timeframe, of achieving regulatory approval has limited the development of improved GE animal varieties by public sector scientists and small companies.^22^ Despite nearly 35 years of research and the approval of multiple GE crop varieties, not a single transgenic food animal has successfully made its way to U.S. consumers. The only GE animal to ever be approved for food purposes via the new animal drug provisions of the FD&C Act, the AquAdvantage GE salmon, was mired in regulatory limbo for years,^23^ and incurred development and regulatory costs running into the tens of millions of dollars. And despite its improved feed conversion efficiency and more efficient utilization of dietary protein^24,25^ and finally obtaining FDA regulatory approval in November 2015,^26^ more than a quarter of a century and many generations since the founder fish was first developed by academic researchers^27^ in Canada in 1989, the company is currently prevented from importing and selling its product in the United States. This is due to a provision in the U.S. government’s budget for fiscal year 2017, introduced by Alaskan Senator Lisa Murkowski, which instructs the FDA to forbid the sale of the transgenic salmon until it has developed a program to inform consumers that they are buying a genetically engineered or bioengineered product.^28^

The 2017 FDA draft guidance on gene edited animals doubles down on this approach by proposing to regulate all genomic alterations introduced into animals by gene editing as new animal drugs. This includes many of the same nucleotide insertions, substitutions, or deletions that could be obtained using conventional breeding. No longer is it the presence of a transgenic rDNA construct that triggers mandatory premarket FDA regulatory oversight prior to commercial release, but rather it is the presence of any “intentionally altered genomic DNA” in an animal that initiates oversight. This does not make sense from a public safety perspective as the fact that an alteration is intentional does not have any relationship to product risk.^3^ If genome edited livestock will be required to comply with the same regulatory standards as genetically engineered animals, then companies with the resources to cope with such regulatory burdens are likely to be favored.^22^

Mandating premarket regulatory approval for deletions, mutations, and the conversion of one naturally-occurring allele to another naturally-occurring allele in the same species (cisgenic) that could have been obtained using conventional breeding runs counter to the OSTP policy, the recommendations of the U.S. National Academies of Sciences, Engineering, Medicine 2016 report^29^ which recommended a “product not process” regulatory trigger approach, the stated USDA approach to the regulation of gene edited plants, and is also out of step with decisions being made by other regulatory agencies in a number of countries around the world, with implications on global trade.

At the end of the day, food animals with intentional genomic alterations produce food, and if the food they produce is not biologically active via an oral route of administration it does not make sense to regulate these intentional genomic alterations as drugs.^30^ Referring to a DNA sequence variant as a “drug” is likely to confuse or frighten consumers who might infer that there are biologically active substances in their food. A DNA alteration is not a drug, but rather part of the genetic code uniquely associated with any organism. Through its natural function within a cell, DNA controls how an organism grows and its unique form and function. The phenotype will ultimately be determined by the interaction of an organism’s genomic DNA sequence and the environment in which it lives. We do not regulate the millions of spontaneous genetic variations that are in our food because DNA is generally regarded as safe to consume,^31^ and it is a routine ingredient of food obtained from any species, irrespective of its sequence.

## Why does this matter?

Given that no single GE food animal product is currently commercially available in the United States, animal breeders are perhaps the group most aware of the chilling impact that regulatory gridlock can have on the deployment of potentially valuable breeding techniques. This would suggest that the FDA’s regulatory approach is unfit for purpose as there does not appear to be a viable path for safe products to come to market.^30,32^ In addition, the historical focus on the potential effects of an intergeneric rDNA as a new animal drug is not applicable to gene editing strategies that result in no transgenic DNA. Examples of GE animals include disease-resistant animals,^33–38^ products with improved quality attributes^39^ and/or lacking common allergens,^40^ and production animals with reduced environmental footprints.^41^ The prohibitive cost of achieving regulatory approval has limited the development of improved GE animal lines by public sector scientists and small companies.^42^ Delaying or preventing the use of this technology in animal breeding programs is associated with very real opportunity costs in terms of foregone genetic improvement.^32,43^

The advent of gene editing offered an opportunity to rethink the regulatory approach to the products of modern biotechnology, and a number of authors have argued that the trigger for regulatory review should be novel product hazards/risks, if any, weighed against the resulting benefits.^30,32,42,44–49^ Researchers are already working on a range of beneficial gene edited food animal applications^14,45,50^ addressing important zoonotic disease and animal welfare traits such as dehorning and castration. Great potential exists to use gene editing to translate the understandings that have been derived from the significant public investment in food animal genome sequencing projects^51–56^ into useful practice.^2^ Some of the most well-known of these food animal applications include disease resistant animals such as pigs carrying a deletion that provides resistance to the devastating porcine reproductive and respiratory syndrome (PRRS) virus,^57–59^ and dairy cows that carry a naturally-occurring bovine allele for the *POLLED* gene,^60^ which means that they do not grow horns and are therefore spared the painful process of their physical removal. Both of these examples benefit animal health and welfare which are improvements that tend to be favorably viewed by the public.^61^

In neither of these examples is there a transgenic rDNA combination of genetic material, and yet under the draft 2017 FDA guidance, both of these examples will be subject to a mandatory regulatory review prior to commercial release under the auspices of the new animal drug provisions of the FD&C Act. In one case the absence of DNA (a gene deletion) is the “drug”, a drug that will be transmitted to all descendants of that animal via reproduction. In the other case the drug is a naturally-occurring 212 bp DNA sequence^62^ that is not otherwise regulated and that we routinely consume in products from beef cattle. This sequence will be regulated as a drug when edited into a dairy cattle genomic background solely based on the process used to produce the variant (Fig. 1). Regulatory oversight should be commensurate with risk meaning that products that pose no/low risk should have minimal regulatory oversight, while those that pose high risks should face extensive regulatory scrutiny. Additionally, regulation should be even-handed, meaning products with the same level of risk should receive equal scrutiny irrespective of the process used to produce them.

A large breeding company, Genus PLC, has announced it will take the PRRS-resistant gene deletion pigs through the FDA’s regulatory review process. Given the importance of this disease to the global pig industry, and the resources available to a large company like Genus, this is perhaps a reasonable decision. However, academic researchers and small companies face a dramatically different situation. The draft guidance suggests the need for genotypic and phenotypic durability studies over multiple generations, including, where feasible, data on inheritance from at least two generations, preferably more, and recommends that at least two of the sampling points be from non-contiguous generations (e.g., F1 and F3). Further, the draft guidance recommends that all surplus investigational animals and their biological products be disposed of by incineration, burial, or composting. Multigenerational studies with large food animals like cattle take years and are beyond the resources of most academic laboratories, especially if the investigational animals have to be incinerated rather than sold for food purposes. And while these requirements might make some sense in the context of animals expressing a pharmaceutical protein (i.e., an actual drug), they make little sense in the context of a DNA deletion or a naturally-occurring allele in food. How can the absence of small piece of DNA rationally be considered a drug?

Another requirement outlined in the draft guidance is “full characterization of the site of intentional alteration, any unintended alterations (e.g., off-target alterations, unanticipated insertions, substitutions, or deletions)”. It is further recommended that researchers evaluate whether there are any unintended interruptions of coding or regulatory regions. Given the millions of natural genetic variations that exist between any two individuals, and the observation that unanticipated insertions, substitutions, or deletions occur every meiosis,^12^ it is unclear how this requirement can be fulfilled in a way that differentiates between unintended alterations and spontaneously-occurring insertions, substitutions, deletions, and other unanticipated naturally-occurring alterations as shown in Fig. 2. The analyses and interpretation of whole-genome sequencing data can also be inconsistent among research groups, making it difficult to standardize from a regulatory perspective.^13^ Animals produced by conventional breeding methods are not routinely evaluated for unintended effects at the molecular level.^3^

Such heavy regulatory burdens would be anticipated to be associated with very high-risk products. And yet it is actually difficult to come up with a unique hazard (harm), let alone risk (probability of harm), associated with animals that could otherwise have been developed through traditional breeding techniques based solely on the fact that they carry intentional genomic alterations introduced by gene editing. The draft guidance divides food safety risk into two overall categories. The first is examining whether there is any direct toxicity, including allergenicity, via food consumption “of the expression product of the article”. And while this risk might be associated with the expression product of a transgene, it again makes little sense for animals that could otherwise have been developed through traditional breeding techniques. The fact that such studies are not required or performed on animals developed through traditional breeding techniques makes this requirement disproportionate from a regulatory perspective, especially given the low historical food safety risks that have been associated with conventional animal breeding.

The second category of purported food safety risk requires performing studies to identify indirect toxicity associated with any biologically relevant changes to the physiology of the animal, and to determine if the composition of edible tissues from the animals whose genomes have been intentionally altered differs from conventional products. This high level of regulatory scrutiny is perhaps intended to assuage public fears or placate opponents. However, the disproportionate regulatory burden for products that could have been achieved using conventional breeding will likely disincentivize the use of gene editing in U.S. food animal breeding programs, and result in the choice of less-efficient processes (e.g., introgression) to introduce useful genetic variation purely for their immunity to premarket regulatory hurdles.

It should be emphasized that existing breeding programs already thoroughly phenotype selection candidates, looking for undesired phenotypes or negative correlations that might exist between important selection objectives. For example, one large breeding company phenotypically evaluates broiler selection candidates for 56 traits, and more than 50% of these traits are measures of fitness and health. These traits include skeletal and leg abnormalities, various physiological measures of heart and lung functions, and specific causes of mortality.^63^ As with plant breeding, “off-types” do not advance to become parents of the next generation. Selection pressure for viable and healthy individuals is intense at the pedigree level of the breeding pyramid, where each successful candidate can potentially give rise to millions of descendants.^64^

Finally, there is the incompatibility of the proposed FDA regulatory approach with the structure of animal breeding programs. A number of lines and/or breeds, and therefore multiple “founder” animals, will likely need to be edited for the exact same trait. For example, in the broiler chicken industry, most primary breeders cross multiple different breeding lines to service different industry needs. And in the case of the dairy industry, the *POLLED* allele would need to be introduced into horned breeds (e.g., Holstein and Jersey). And to prevent genetic inbreeding and loss of diversity, the same modification will often need to be introduced into numerous elite artificial insemination bulls.^65^

The FDA draft guidance suggests that each new animal drug application would generally only cover animals derived from a single alteration event. If each individual edited animal is required to go through a multigenerational mandatory premarket regulatory evaluation prior to commercialization, then there will be a fundamental disconnect between the proposed U.S. regulation of gene edited animals, and the realities of genetic improvement programs where future parents are selected from every subsequent generation because those animals are genetically superior to their parents.

**Fig. 3:**
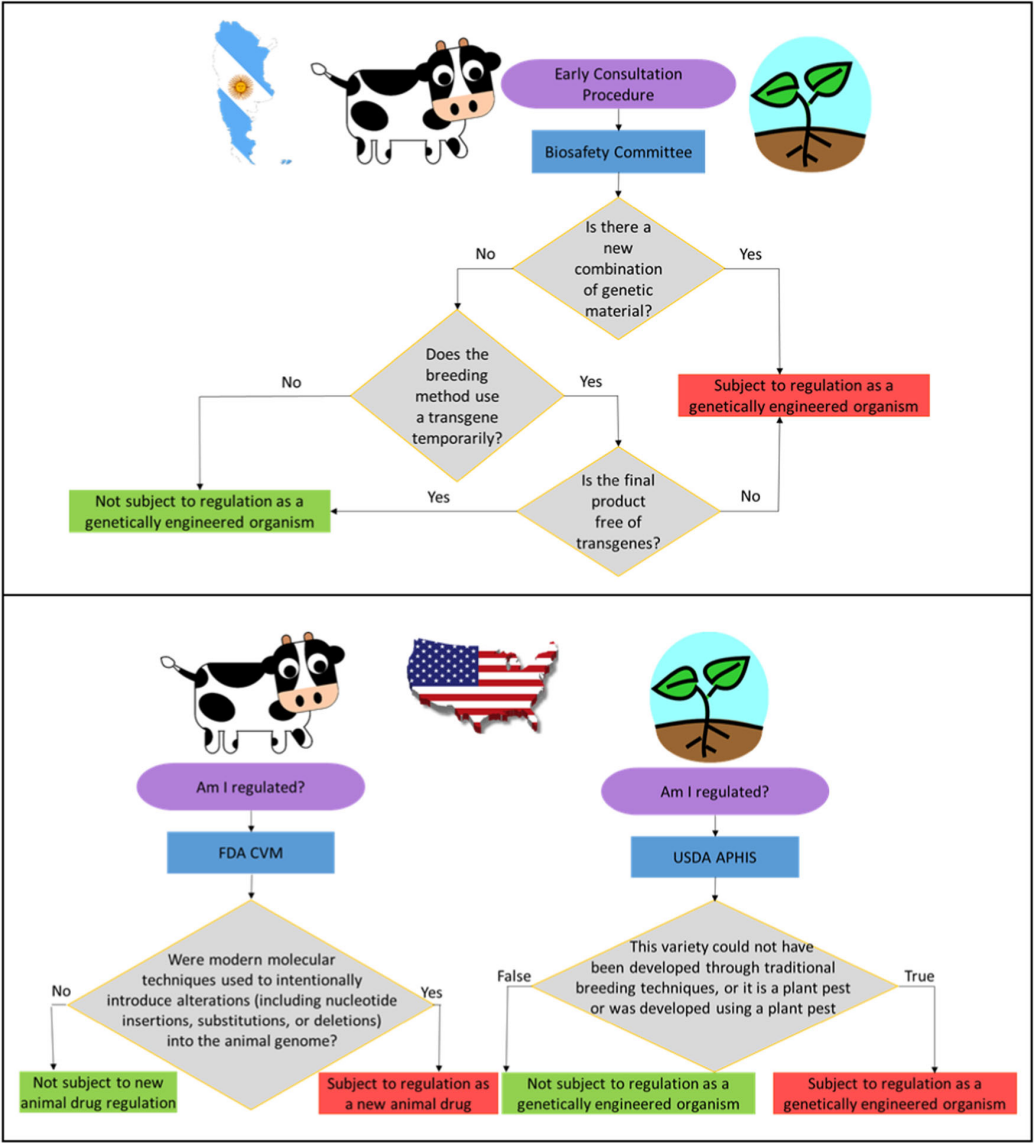
Flow map contrasting proposed regulation of genome-edited food animal species applications in (**a**) Argentina (modified from Whelan and Lema^68^), and (**b**) proposed United States regulation. Modified from Van Eenennaam (2018)^49^

It is no accident that gene edited food animal applications are moving to countries with novel product-based regulatory triggers for gene edited animals. Argentina was the first country to publish their proposed approach to the regulation of gene edited organisms.^66^ They plan to regulate plants and animals in the same way, and the trigger for regulation will be whether plants or animals carry a “novel combination of genetic material” (i.e., intergeneric). Those that do will be considered a “GMO” under Argentine law, and those that do not will not trigger additional regulatory oversight irrespective of the use of modern biotechnologies or rDNA techniques in the breeding process (Fig. 3). Canada, the only country that has ever allowed the commercial sale of a GE animal, the AquAdvantage GE salmon approved in 2016,^28^ has a product-based regulatory system triggered by product novelty, regardless of the breeding technique that was used to obtain the plant or animal end product.^47^

The gene-editing company Recombinetics, has announced an alliance with bovine genetics provider Semex in Canada to introgress the naturally-occurring *POLLED* allele into their elite dairy genetics using gene editing.^67^ In October 2018, the National Technical Biosafety Commission (CTNBio) in Brazil concluded that semen from an edited bull carrying the P_C_ *POLLED* intraspecies allele substitution^60^ would not be considered a “genetically modified organism” under their regulatory schema.^68^ Likewise, Argentina's National Advisory Commission on Agricultural Biotechnology (CONABIA) has evaluated proposed gene edited animals that do not contain any foreign DNA or a new combination of genetic material and judged them to be exempt from GM regulation. These include gene edited applications in fish (tilapia), cattle, and horses. In the absence of regulatory harmony, breeders in some countries will have the ability use gene editing in agricultural breeding programs, while those in other countries will not, resulting in disparate breeder access to these tools, and ultimately the potential for trade disruptions.

The FDA’s draft “Guidance for Industry #187” entitled “Regulation of Intentionally Altered Genomic DNA in Animals” is not fit for purpose as it relates to food animals that could otherwise have been developed through traditional breeding techniques. We reject the idea that intentional genomic DNA alterations should be regulated as a veterinary drug in food animals, and consider that the proposed approach will thwart the development of genetic approaches by public sector researchers and small companies to use gene editing to solve zoonotic disease and animal welfare problems in the United States. We further support the call made by scientists at the 2019 Plant and Animal Genome meeting (https://www.gopetition.com/petitions/harmonize-us-gene-edited-food-regulations.html) that the U.S. regulatory system should be harmonized so that both plants and food animals that could otherwise have been developed through traditional breeding techniques are not subject to additional premarket regulatory requirements based solely on the fact that intentional genomic alterations were introduced using modern biotechnologies or rDNA techniques in the breeding process.
